# Fluvastatin inhibits AGE-induced cell proliferation and migration via an ERK5-dependent Nrf2 pathway in vascular smooth muscle cells

**DOI:** 10.1371/journal.pone.0178278

**Published:** 2017-05-22

**Authors:** Ae-Rang Hwang, Jung-Hwa Han, Jae Hyang Lim, Young Jin Kang, Chang-Hoon Woo

**Affiliations:** 1 Department of Pharmacology, Yeungnam University College of Medicine, Daegu, Republic of Korea; 2 Smart-Aging Convergence Research Center, Yeungnam University College of Medicine, Daegu, Republic of Korea; 3 Department of Microbiology, Ewha Womans University School of Medicine, Seoul, Republic of Korea; University of South Alabama Mitchell Cancer Institute, UNITED STATES

## Abstract

Advanced glycation endproduct (AGE)-induced vascular smooth muscle cell (VSMC) proliferation and reactive oxygen species (ROS) production are emerging as important mechanisms of diabetic vasculopathy, but little is known about the molecular mechanism responsible for the antioxidative effects of statins on AGEs. It has been reported that statins exert pleiotropic effects on the cardiovascular system due to decreases in AGE-induced cell proliferation, migration, and vascular inflammation. Thus, in the present study, the authors investigated the molecular mechanism by which statins decrease AGE-induced cell proliferation and VSMC migration. In cultured VSMCs, statins upregulated Nrf2-related antioxidant gene, NQO1 and HO-1, via an ERK5-dependent Nrf2 pathway. Inhibition of ERK5 by siRNA or BIX02189 (a specific ERK5 inhibitor) reduced the statin-induced upregulations of Nrf2, NQO1, and HO-1. Furthermore, fluvastatin was found to significantly increase ARE promoter activity through ERK5 signaling, and to inhibit AGE-induced VSMC proliferation and migration as determined by MTT assay, cell counting, FACS analysis, a wound scratch assay, and a migration chamber assay. In addition, AGE-induced proliferation was diminished in the presence of Ad-CA-MEK5α encoding a constitutively active mutant form of MEK5α (an upstream kinase of ERK5), whereas depletion of Nrf2 restored statin-mediated reduction of AGE-induced cell proliferation. Moreover, fluvastatin suppressed the protein expressions of cyclin D1 and Cdk4, but induced p27, and blocked VSMC proliferation by regulating cell cycle. These results suggest statin-induced activation of an ERK5-dependent Nrf2 pathway reduces VSMC proliferation and migration induced by AGEs, and that the ERK5-Nrf2 signal module be viewed as a potential therapeutic target of vasculopathy in patients with diabetes and complications of the disease.

## Introduction

Hydroxy-3-methylglutaryl coenzyme A reductase inhibitors (statins) are potent inhibitors of cholesterol biosynthesis and are widely used to reduce serum cholesterol levels in hyperlipidemic patients [1]. However, recent reports have shown statins also ameliorate cardiovascular disorders, and remarkably, have preventative effects on cardiovascular diseases [2]. In addition, statins have been reported to improve endothelial dysfunction by increasing nitric oxide availability, to inhibit proliferation and inflammatory responses, and to stabilize atherosclerotic plaque [3, 4].

Extracellular signal-regulated kinase 5 (ERK5) is an atypical member of MAPK family, and reportedly, regulates endothelial integrity and protects against vascular dysfunction and cardiovascular diseases in rodent models. On the other hand, MEK5, an upstream kinase of ERK5, plays critical roles in cell proliferation, migration, and differentiation [5]. Transcription factor nuclear factor-erythroid 2-related factor 2 (Nrf2) is an important regulator of cellular oxidative stress [6, 7], and under homeostatic conditions, binds to Kelch-like ECH-associated protein 1, and is subsequently degraded via the proteasome system or stored in cytoplasm [8]. In the presence of oxidative stress, Nrf2 translocates to the nucleus, where it forms Nrf2/small Maf heterodimer, which binds specifically to antioxidant response elements (AREs), and activates the gene expressions of antioxidant proteins, such as, NAD(P)H:quinone oxidoreductase-1 (NQO1), and heme oxygenase-1 (HO-1) [9–15]. Nrf2 has also been reported to play an atheroprotective roles by regulating antioxidant genes in the cardiovascular system [16]. Cellular redox balance is tightly controlled by various antioxidant systems, and in VSMCs, ROS activates the Nrf2 signaling pathway, which in turn, induces anti-atherosclerotic gene expression [17], and it has been reported Nrf2-activating drugs decrease VSMC proliferation and migration by antioxidant gene expression [18, 19]. Furthermore, studies have demonstrated ERK5 is a molecular target for regulating laminar blood flow-mediating Nrf2-dependent gene expression and suggested it may have significant therapeutic potential for the treatment of atherosclerosis [20].

It was recently shown advanced glycation endproducts (AGEs) and their receptor-ligand interactions play key roles in neointimal formation after vascular injury [21, 22]. AGEs are known to be formed in diabetes and to promote inflammation via specific receptors on endothelial cells [23, 24], and to mediate pro-inflammatory responses and cell proliferation through the NFκB signaling pathway [25, 26]. We hypothesized that statin might decrease AGE-induced proliferation and migration via ERK5-Nrf2-dependent gene regulation in VSMCs, and thus, we sought to identify the molecular mechanism responsible for the reductions in AGE-induced cell proliferation and VSMC migration by statins.

## Materials and methods

### Reagents and antibodies

Fluvastatin, pitavastatin, and BIX02189 (a specific inhibitor of ERK5) were purchased from Selleck Chemicals (Houston, TX) [27]. AGE-BSA was obtained from Calbiochem (Darmstadt, Germany), and MTT reagents were purchased from Amresco (Solon, Ohio). Antibodies were purchased from the following vendors: ERK1/2 (#9102, anti-rabbit), ERK5 (#3372, anti-rabbit), phosphor-ERK1/2 (#9106, anti-rabbit) and phospho-ERK5 (#3371, anti-rabbit) from Cell Signaling Technology (Danvers, MA), Nrf2 (sc-13032, 200 μg/ml, anti-rabbit), NQO1 (sc-32739, μg/ml, anti-mouse), CDK4 (sc-260 C-22, 100 μg/ml, anti-rabbit), p27 (sc-528 C-19, 100 μg/ml, anti-rabbit) and HA (anti-rabbit) from Santa Cruz Biotechnology (Santa Cruz, CA), HO-1 (ADI-SPA-895, 1 mg/ml, anti-rabbit) from Enzo lifesciences, cyclin D (06–137, 1 mg/ml, anti-rabbit) from Millipore and tubulin (anti-mouse) from Sigma (St. Louis, MO).

### Cell culture and treatment conditions

Sprague-Dawley rats were anesthetized with ketamine and xylazine. Primary rat vascular smooth muscle cells (VSMCs) were isolated from rat thoracic aorta. The cells were processed using a 1 mm chop setting in a 10 cm culture dish, and cultured with 50% FBS-DMEM with 1% antibiotics-antimycotics in a CO2 incubator. VSMCs were maintained in DMEM supplemented with 10% fetal bovine serum (FBS), 50 U/mL penicillin, and 50 μg/mL streptomycin at 37°C in a 95% air-5% CO_2_ atmosphere. VSMCs from passages 4–7 were cultured for 9–24 h in the presence of 5μM fluvastatin with or without 10 μg/ml AGEs. Cells were harvested at indicated time points. All animal experiments were handled in accordance with the protocol approved by the Institutional Animal Care and Use Committee at Yeungnam University College of Medicine, Daegu, *Republic of Korea*.

### Western blotting analysis

Cells were lysed with radioimmunoprecipitation assay (RIPA) lysis buffer supplemented with 1 mM phenylmethylsulfonyl fluoride (PMSF) and 0.01 mM protease inhibitor cocktail (PIC), and lysates were incubated on ice for 15 min and centrifuged at 15,000 × g for 10 min at 4°C. Protein concentrations were determined using a Bradford assay. Proteins were separated by SDS-PAGE and transferred to polyvinylidene difluoride (PVDF) membranes, which were then immunoblotted with primary antibodies (1:1,000 dilution) followed by corresponding secondary antibodies (1:4,000 dilution). Signals were visualized using electrochemiluminescence (ECL) detection reagents (Millipore, Temecula, CA), according to the manufacturer’s instructions.

### Quantitative real time RT-PCR (qRT-PCR)

The mRNA expressions of Nrf2 targeted genes were determined by qRT-PCR as described previously [7]. Briefly, total RNA was isolated with TRIzol, and reverse transcription was conducted using TaqMan reverse transcription reagents, according to the manufacturer’s instructions. qRT-PCR was conducted using 1 μL of template cDNA and Power SYBR Green in an ABI PRISM 7500. Quantification was performed using the efficiency-corrected ΔΔCq method. The primers used to amplify DNA sequences were as follows: NQO1, forward 5′-TTACTATGGGATGGGGTCCA-3′ and reverse 5′-TGCCAAAACTGTTCACCAAA-3′, Nrf2, forward 5′-AAACCACCCTGAAAGCACAG-3′ and reverse 5′-AGTGTTCTGGTGATGCCACA-3′; and GAPDH, forward 5′-GGAGCCAAAAGGGTCATCAT-3′ and reverse 5′-GTGATGGCATGGACTGTGGT-3′. PCR conditions were as follows: preliminary denaturation at 50°C for 2 min, followed by 95°C for 10 min, 95°C for 15 s, and 60°C for 1 min.

### Small interfering RNA (siRNA)

VSMCs were transiently transfected with 50 or 100 pM of control or specific siRNAs against Nrf2 or ERK5 using Lipofectamine 2000 reagent (Invitrogen) according to the manufacturer’s instructions. The targeting sequences of siRNAs were as follows: rat Nrf2 siRNA (5’-CAAACAGAAUGGACCUAAAdTdT-3’); rat/mouse ERK5 siRNA(5’-GAAAGGGTGCGAGCCTATAUU-3’). Non-specific control siRNA was purchased from Bioneer and used as a negative control. Cells were harvested 48–72 h after siRNA transfection, and mRNA expression levels were measured.

### MTT assay

AGE-induced proliferation was quantified using a MTT assay. Briefly, VSMCs were cultured on 24-well plates and when ~80% confluent, medium was replaced with serum free DMEM. Cells were then pretreated with BIX02189 (2 μM) and stimulated with fluvastatin (5 μM) for 24 h. MTT reagents were added for 4 h at 37°C the removed by washing with PBS, and eluted with DMSO. Proliferation was measured using a microplate reader (Biorad) at 570 nm.

### Reporter gene assay

A luciferase assay was used to determine ARE promoter activity. The resultant construct was co-transfected with pRL-tk vector containing Renilla luciferase reporter gene into cultured VSMCs using the lipofectamine 2000 method and Plus transfection reagent. Cells were lysed at 24 h post-transfection, and Firefly-to-Renilla luciferase activity ratios in lysates were measured with dual-luciferase assay kit (Promega) to evaluate ARE promoter activity.

### Flow cytometric analysis

Cells (1×10^5^) were trypsinized, fixed in 95% ethanol, and stained with propidium iodide (PI) (50 μg/ml) for 30 min at 37°C. PI stained cells were filtered using a 5 ml polystyrene round bottom tube fitted with a cell-strainer cap prior to flow cytometry. All flow cytometry measurements were obtained using a FACSCalibur (Becton Dickinson, San Jose, CA). Cell cycle analysis was performed using Cell-Quest pro-software.

### Wound scratch assays and migration chamber assays

VSMC cells were cultured until >90% confluent in 6 well dishes, and then medium was replaced with serum free DMEM overnight. Wounds were made with a sterile 200 μL pipet tip by drawing a line through cells perpendicular to the line above. Cells were then pretreated with BIX02189 (2 μM) and stimulated with fluvastatin (5 μM) for 24 h. Finally, images were taken using a phase contrast at 40×. For selective migration assay, transwell system was used. Cells were seeded in the inner chamber and pretreated with BIX02189 (2 μM) in the presence or absence of fluvastatin (5 μM). AGE (10 μg/ml) were added to the lower for 12 h. After fixing, cells were visualized by crystal violet staining. Unmigrated cells were scraped off and then migrated cells were counted under a light microscope.

### Statistics

Results shown in the bar graph are means ± SDs. The significances of differences were determined using ANOVA and Student’s *t* test. *P* values of < 0.05 were considered significant.

## Results

### Inhibition of ERK5 reduced statin-induced expressions of Nrf2 and NQO1 in VSMCs

**Fig 1:**
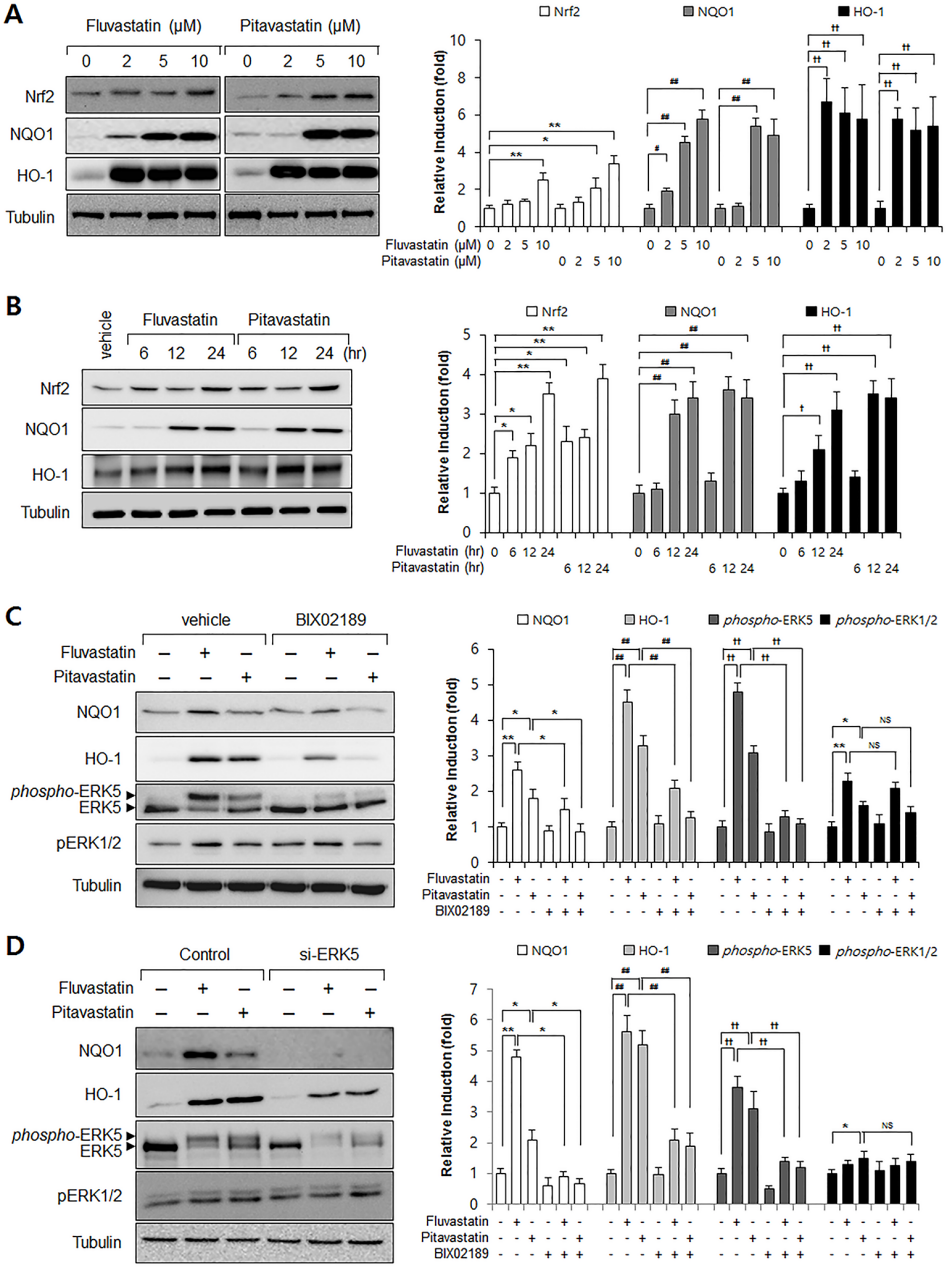
The involvement of ERK5 in statin-induced Nrf2 signaling in VSMCs. (A-B) Western blot analysis of Nrf2, NQO1, and HO-1 in statin-treated VSMCs. Cells were exposed to fluvastatin and pitavastatin for 24 h at the indicated dosages. In addition, protein samples were refined from cultured VSMCs treated with fluvastatin (5 μM) or pitavastatin (5 μM) for the indicated times. (C) Western blot analysis of ERK5, NQO1, HO-1, phospho-ERK1/2, and phospho-ERK5 in BIX02189 treated VSMCs. Protein samples were obtained from cultured VSMCs treated with 5 μM fluvastatin or 5 μM pitavastatin for 24 h. (D) VSMCs were transfected with control or ERK5 siRNA (50 pM) for 30 h and then treated with 5 μM fluvastatin for 24 h. Protein levels of NQO1, HO-1, ERK5 and tubulin were determined by Western blotting with specific antibodies. In addition, phosphorylation levels of ERK1/2 and ERK5 were determined by immunoblotting with specific antibodies. Bar graphs present the densitometric quantification of Western blot bands. Results are representative of three independent experiments. *, #, †, p<0.05; **, ##, ††, p<0.01 compared with control. NS indicates not significant.

To investigate the effect of statins on the Nrf2 antioxidant system, we assessed Nrf2, NQO1, and HO-1 protein levels in VSMCs. As shown in Fig 1, fluvastatin and pitavastatin strongly increased Nrf2 and NQO1 expressions in dose- and time-dependent manner (Fig 1A and 1B). To examine the role of ERK5 in Nrf2 antioxidant system induction by statins, we used an ERK5 inhibitor and ERK5 specific siRNA. Inhibition of ERK5 was found to markedly attenuate fluvastatin-induced NQO1 and HO-1 expression (Fig 1C and 1D). Interestingly, statin-induced phosphorylation levels of ERK1/2 were not affected by ERK5 inhibitor and siRNA (Fig 1C and 1D), suggesting the specific role of ERK5 in statin-induced Nrf2 signaling in vascular smooth muscle cells.

**Fig 2:**
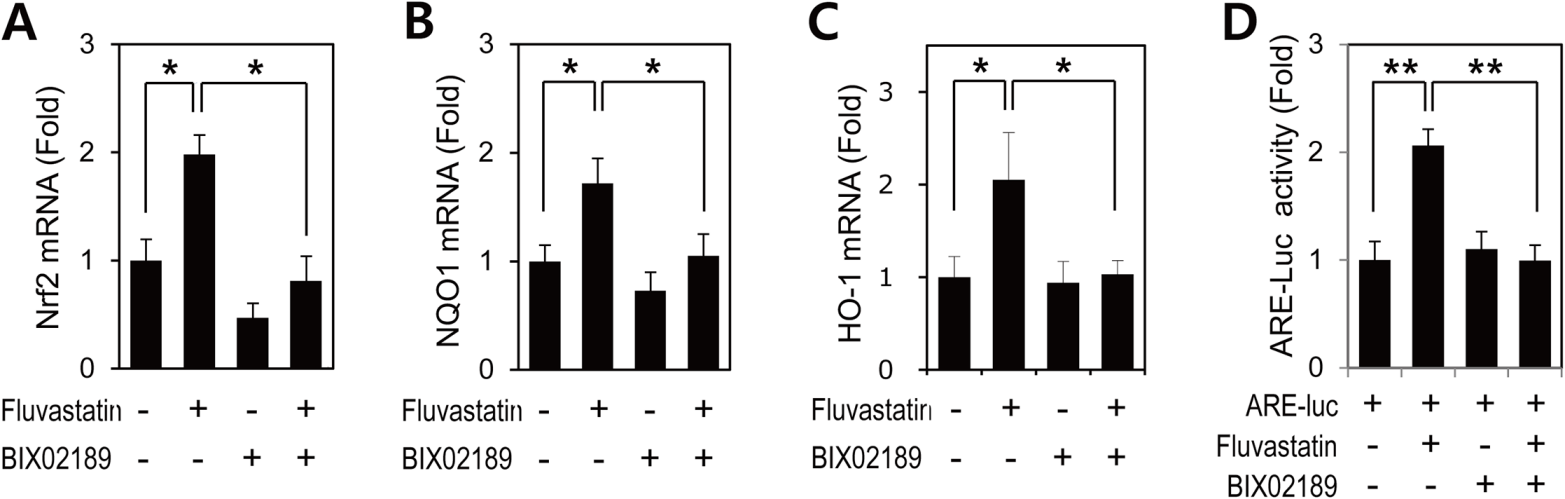
The involvement of ERK5 in fluvastatin-induced Nrf2 signaling in VSMCs. (A-C) Quantitative RT-PCR analysis of the mRNA expressions of Nrf2, NQO1, and HO-1 in VSMCs treated with fluvastatin. VSMCs were treated with BIX02189 (2 μM) for 1 h and then incubated with 5 μM fluvastatin for 6 h. qRT-PCR analysis was performed in triplicate. Results are representative of three independent experiments. *, p < 0.05. (D) VSMCs were co-transfected with pARE and pRL-tk and then stimulated with 5 μM fluvastatin for 24 h in the presence or absence of BIX02189 (2 μM). Promoter activity was measured by using a Dual-Luciferase reporter assay kit and a GloMax 20/20 luminometer. Transfection efficiency was normalized versus *Renilla* luciferase activity derived from pRL-tk construct. Reporter assay was performed in triplicate. Results are presented as the means ± SDs of three independent experiments. **p < 0.01.

Next, we studied the effect of fluvastatin on the mRNA expressions of Nrf2, NQO1, and HO-1 in VSMCs. Consistent with protein levels, fluvastatin significantly induced the mRNA expressions of Nrf2, NQO1, and HO-1 (Fig 2), and ERK5 inhibition suppressed the fluvastatin-induced expressions Nrf2, NQO1, and HO-1 mRNA (Fig 2). These results indicated that ERK5 contributed to the fluvastatin-induced Nrf2 antioxidant genes in VSMCs.

### Inhibition of ERK5 attenuated fluvastatin-induced ARE promoter activity in VSMCs

Activated Nrf2 translocates to the nucleus and then binds specifically to ARE in the promoters of target genes, such as, NQO1 and HO-1 [8]. Thus, we investigated whether fluvastatin could promote Nrf2 to ARE promoter binding. VSMCs were transfected with the reporter ARE, and ARE promoter activity was measured using a reporter gene assay. ARE promoter activity was found to be enhanced in response to fluvastatin, and to be significantly inhibited by BIX02189 (Fig 2D). These observations suggest that ERK5 is an important regulator of fluvastatin-induced Nrf2 activation and that it does so by regulating transcription.

### Fluvastatin regulated AGE-induced cell proliferation through ERK5-Nrf2 signal module

**Fig 3:**
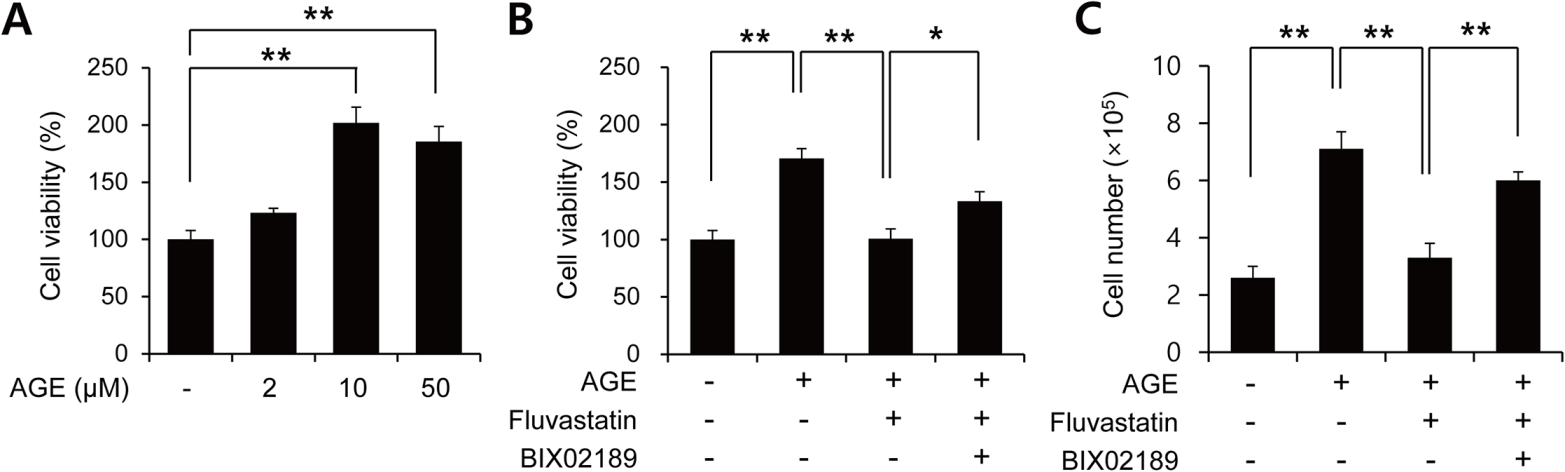
Fluvastatin inhibited AGEs-induced cell proliferation through the ERK5-Nrf2 pathway in VSMCs. (A) Serum starved VSMCs were exposed to the indicated concentrations of AGEs for 24 h. (B) Serum starved VSMCs were pretreated with 5 μM fluvastatin in the presence or absence of BIX02189 (2 μM) for 1 h and then exposed to AGEs (10 μg/ml) for 24 h. Cell viability was determined using a MTT assay. (C) Cell proliferation was determined by using cell counting with same condition. Results are representative of three independent experiments. *, p < 0.05; **, p < 0.01.

**Fig 4:**
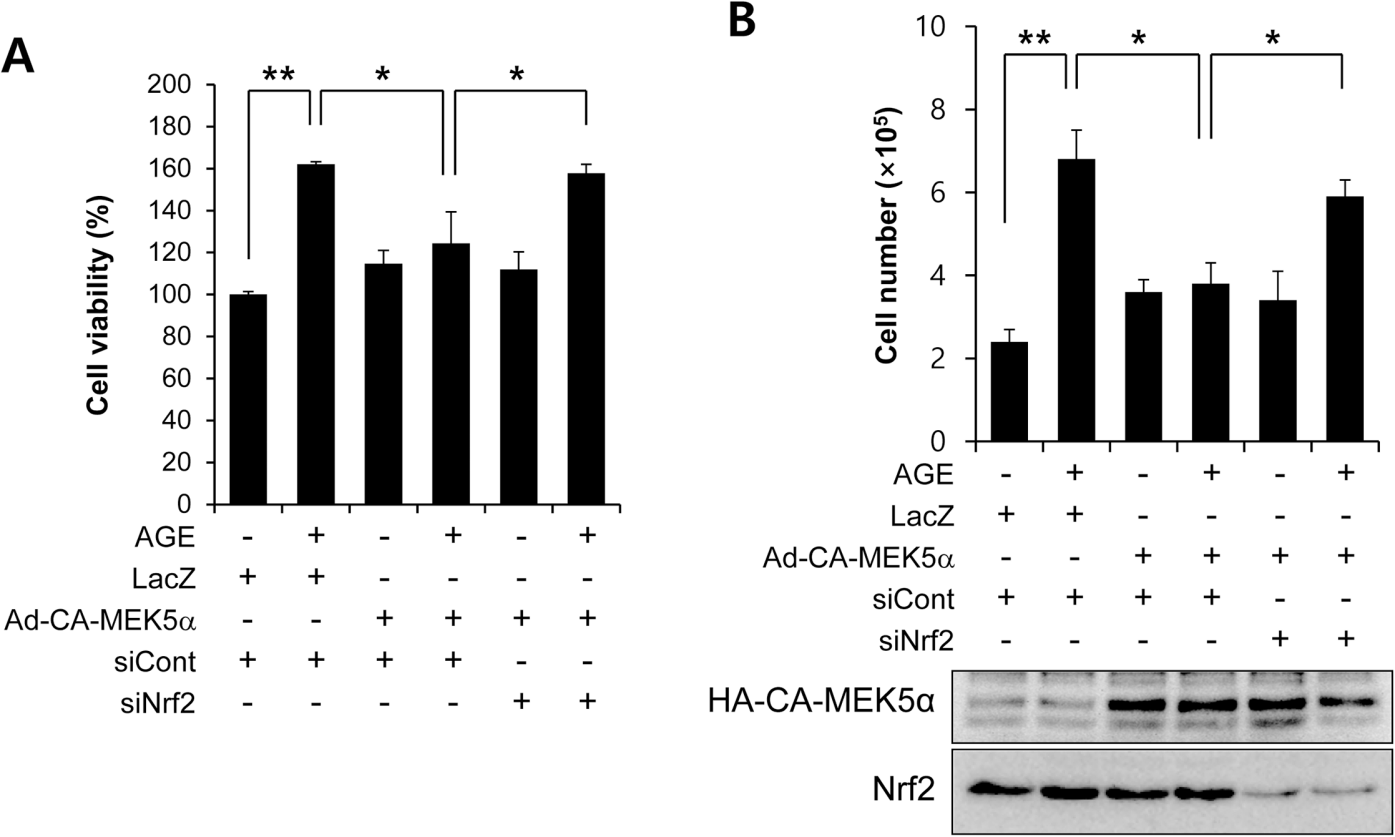
The effect of Nrf2 siRNA on the ERK5 activation-mediated inhibition of AGE-induced VSMC proliferation. (A) VSMC cells were infected with adenovirus encoding LacZ or CA-MEK5α for 48 h, and then exposed to AGEs for 24 h. Cell viability was determined using a MTT assay. Results are presented as the means±SEs of three independent experiments. *, p < 0.05; **, p < 0.01. (B) Cell proliferation was determined by using cell counting with same condition. Results are representative of three independent experiments. **, p < 0.01. Amounts of protein expression were determined by immunoblotting with specific antibodies for HA and Nrf2.

It has been reported that fluvastatin reduces AGE-induced VSMC proliferation [28]. To confirm this effect, VSMCs were treated with AGEs in the presence or absence of fluvastatin and then subject to MTT assay. AGEs were found to dose-dependently induce cell proliferation (Fig 3A), and this was significantly suppressed by fluvastatin (Fig 3B). In addition to MTT assay, we got the similar results with cell counting (Fig 3C). Interestingly, this suppressive effect of fluvastatin was prevented when VSMCs were pretreated with BIX02189, which suggested the involvement of ERK5 in the fluvastatin-mediated inhibition of AGE-induced cell proliferation (Fig 3B and 3C). We also examined whether ERK5 activation could reduce proliferation by using Ad-CA-MEK5α encoding a constitutively active mutant form of MEK5α (an upstream kinase of ERK5). As shown in Fig 4, AGE-induced proliferation determined by both MTT assay and cell counting was significantly diminished in the presence of Ad-CA-MEK5α, and Nrf2 depletion using siRNA restored AGE-induced proliferation. These results indicate fluvastatin inhibited AGE-induced VSMC proliferation through ERK5-Nrf2 signal module.

### Fluvastatin regulated AGEs-induced cell cycle progression through the ERK5-Nrf2 pathway

The cell cycle is controlled by activators (cyclins) and inhibitors (Rb, p16, p21, p27). Among them, cyclin D is a major cell cycle associated cyclin and interacts with four cyclin-dependent kinases (Cdks; Cdk2, 4, 5 and 6). Cyclin D-Cdk4/6 complex accumulation is required for cell cycle progression [29, 30]. On the other hand, p27 inhibits Cdk by binding directly to Cdk-cyclin complex and blocking its protein kinase activity. Statins have been shown to induce cell cycle arrest in various cancer cells, including those of prostate [31] and breast cancer [32], and p53, p21, and p27 proteins are known to regulate VSMC proliferation [33]. Furthermore, it has been demonstrated that statins cause G1 arrest in VSMCs by up-regulating p27 and down-regulating cyclin E [34, 35].

**Fig 5:**
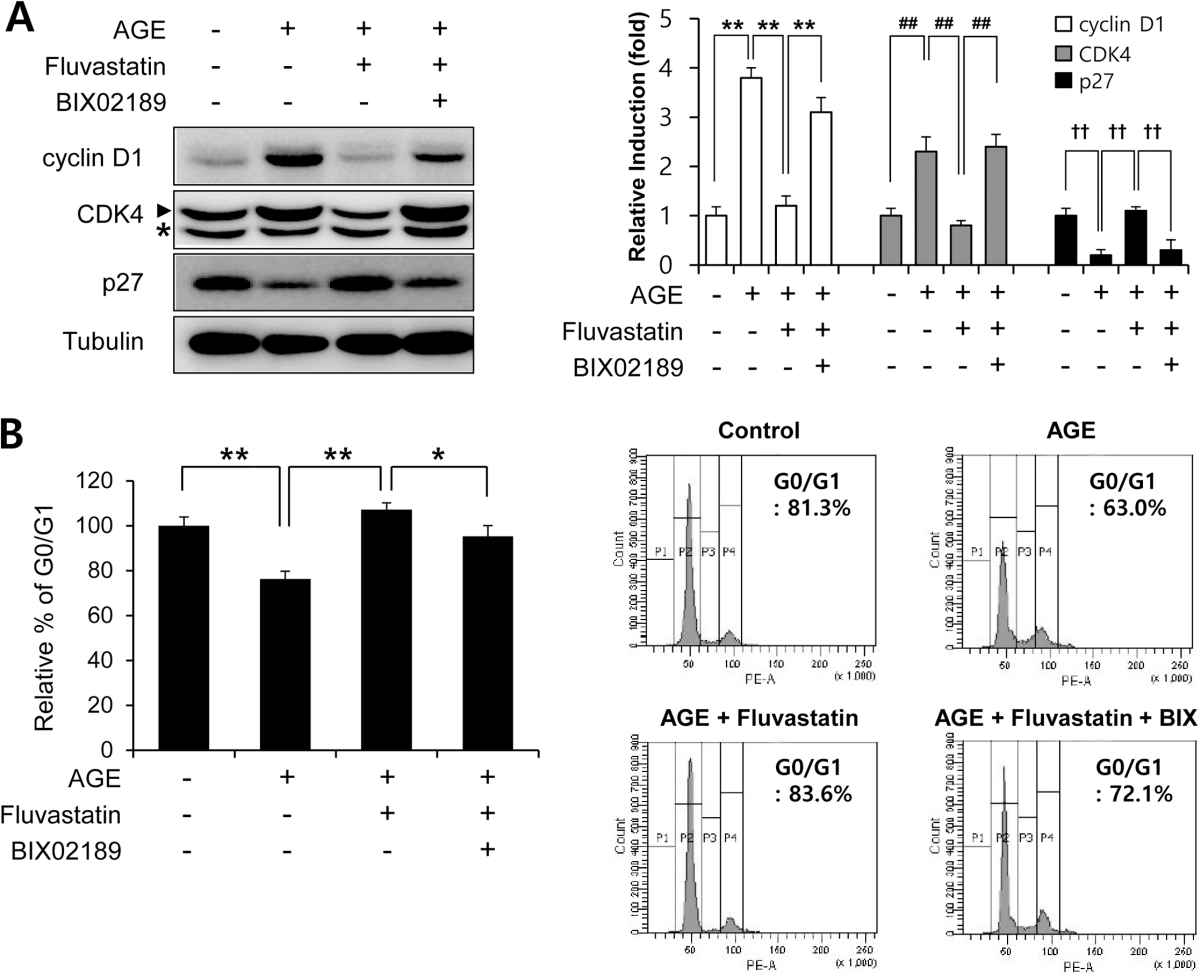
Fluvastatin regulated AGE-induced cell cycle progression through an ERK5 activating pathway in VSMCs. (A) Serum starved VSMCs were pretreated with 5 μM fluvasatin in the presence or absence of BIX02189 (2 μM), and then exposed to AGEs 10 μg/ml AGEs for 24 h. Protein expressions were determined by immunoblotting with anti-cyclin D1, anti-CDK4, anti-p27, and anti-tubulin. Results are representative of three independent experiments. Asterisk indicates a nonspecific band. Bar graphs present the densitometric quantification of Western blot bands. (B) VSMCs were seeded onto 6 well plates at a density of 1×10^4^ cells/ml, pretreated with fluvastatin (5 μM) in the presence or absence of BIX02189 (2 μM), and then exposed to AGEs (10 μg/ml) for 24 h. For cell cycle analysis, cells were then detached, stained with PI, and subjected to flow cytometry. *, p < 0.05; **, p < 0.01 (n = 5).

**Fig 6:**
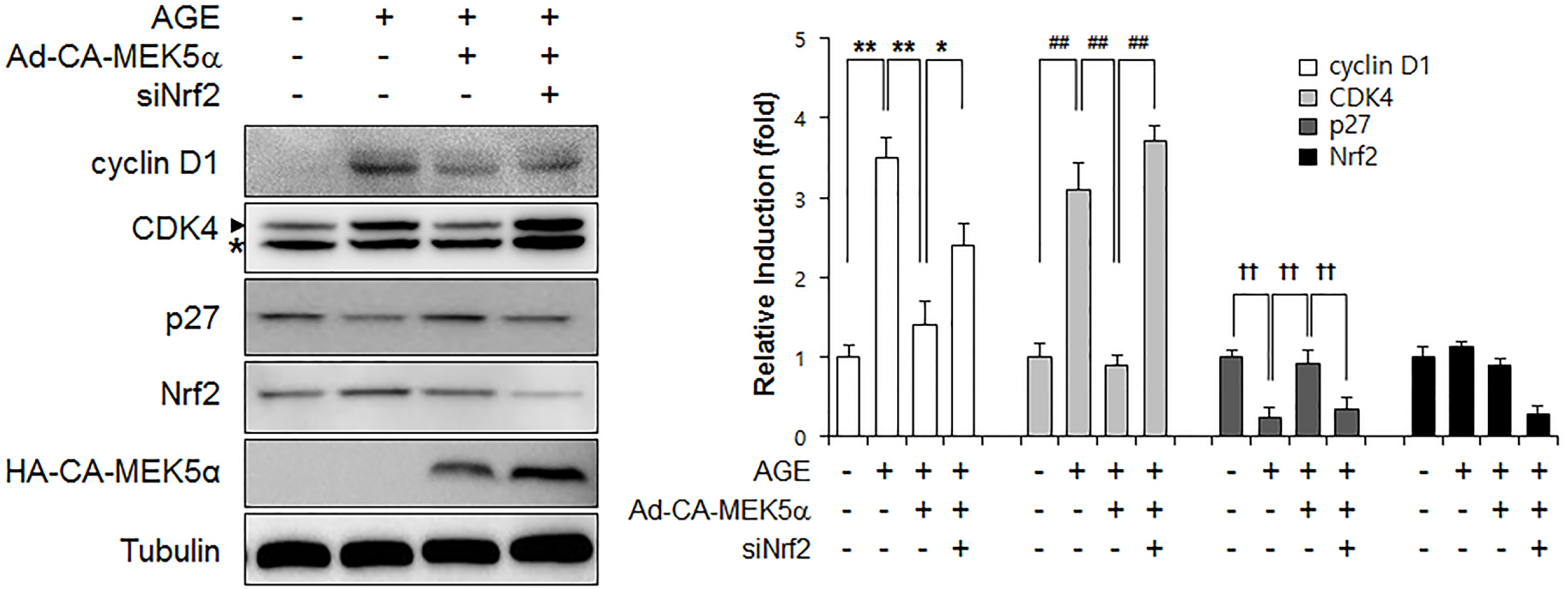
The effect of Nrf2 siRNA on the ERK5 activation-mediated inhibition of AGE-induced cell cycle progression. VSMCs infected with Ad-LacZ or Ad-CA-MEK5α were transfected with siNrf2 for 48 h and then immunoblotted using anti-cyclin D1, anti-CDK4, anti-p27, anti-Nrf2, anti-HA, and anti-tubulin. Results are representative of three independent experiments. Asterisk indicates a nonspecific band. Bar graphs present the densitometric quantification of Western blot bands.

In order to examine the effects of fluvastatin on cell cycle progression at the cell cycle regulatory gene level. We first checked that AGEs increased cyclin D1 and Cdk4 protein levels, but reduced p27 level. Fluvastatin was found to suppress cyclin D1 and Cdk4 protein levels, but to enhance p27 level in VSMCs (Fig 5A), and these effects were prevented by BIX02189 pretreatment. As compared with cells treated with fluvastatin (5 μM) in the presence of AGEs (10 μg/ml), cells in G0/G1phase was significantly reduced by BIX02189 pretreatment (Fig 5B). To investigate whether ERK5 activation induced cell cycle arrest, we pretreated cells with Ad-CA-MEK5α. It was found that the AGE-induced protein expressions of cyclin D1 and CDK4 were markedly reduced in the presence of Ad-CA-MEK5α, whereas depletion of Nrf2 prevented these reductions (Fig 6). These results show fluvastatin blocks VSMC proliferation via an ERK5-Nrf2 pathway and that this leads to cell cycle arrest.

### Fluvastatin regulated AGE-induced cell migration through an ERK5-dependent pathway

**Fig 7:**
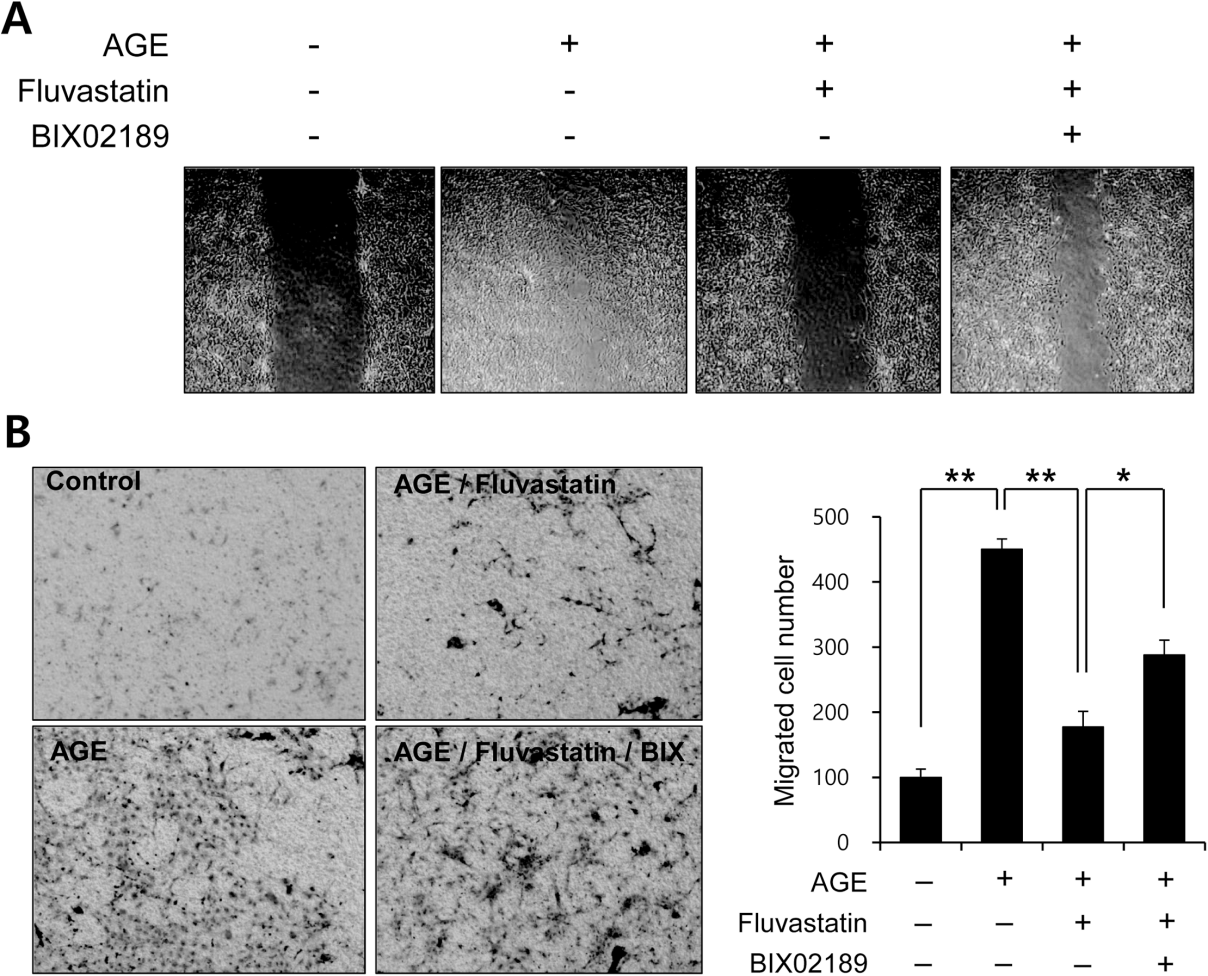
Fluvastatin regulated AGE-induced cell migration through an ERK5-dependent pathway in VSMCs. (A) VSMC cells were cultured until near confluent in 6 well dishes. Cell migration was assessed using a modified scratch assay. VSMCs were pretreated with 5 μM fluvastatin in the presence or absence of BIX02189 (2 μM), and then exposed to 10 μg/ml AGEs for 24 h. Wound areas were visualized using a phase contrast microscope. Results are representative of three independent experiments. (B) For selective migration assay, transwell system was used. Cells were seeded in the inner chamber and pretreated with BIX02189 (2 μM) in the presence or absence of fluvastatin (5 μM). AGE (10 μg/ml) were added to the lower for 12 h. After fixing, cells were visualized by crystal violet staining. Unmigrated cells were scraped off and then migrated cells were counted under a light microscope. Results are representative of three independent experiments. *, p < 0.05; **, p < 0.01.

To determine the functional significance of the fluvastatin-mediated ERK5 activation pathway, we examined the effect of fluvastatin on AGE-induced VSMC migration using both a scratch assay (Fig 7A) and a migration chamber assay (Fig 7B). Fluvastatin significantly suppressed AGE-induced cell migration in response to wound injury, and BIX02189 blocked this inhibitory effect of fluvastatin (Fig 7). Taken together, our results suggest that fluvastatin suppresses AGE-induced cell proliferation and migration by inducing antioxidant genes through the ERK5-dependent activation of the Nrf2 pathway.

## Discussion

Antioxidant defense mechanisms protect cells from excessive oxidative stress [16], and the transcription factor Nrf2 contributes to these processes by regulating the expressions of antioxidant genes. The present study provides evidence that in VSMCs, ERK5 activation increases the expressions of Nrf2 targeted genes involved in cytoprotective responses, and that Nrf2 transcriptional activity is lost when ERK5 expression is depleted or inhibited by ERK5 siRNA or BIX02189 (a biochemical inhibitor of ERK5). To the best of our knowledge, these findings provide first evidence fluvastatin exerts its antioxidant effects through the ERK5-dependent activation of the Nrf2 pathway in VSMCs. Furthermore, the study indicates statins exert their antioxidant effects by increasing the expressions of antioxidant genes in VSMCs, by showing fluvastatin and pitavastatin significantly induce the expressions of antioxidant enzymes (ERK5, Nrf2, NQO1, and HO-1) (Fig 1). Interestingly, we found the up-regulations Nrf2-regulated genes by statin was diminished by ERK5 siRNA or BIX02189 (Fig 1 and Fig 2). It has been reported that statins reduce intracellular ROS in endothelial cells by S-nitrosylating thioredoxin as well as other various effects [18, 24, 36].

A number of recent reports have shown that statins may also have important anti-inflammatory effects, in addition to their effects on lowering plasma lipids [37, 38]. Since inflammation is closely linked to ROS production, the molecular basis of their observed anti-inflammatory effects might be related to their abilities to block ROS production [18, 37, 39]. It has been well established that ROS are related to the development of cardiovascular disease, and many studies support the notion that ROS released from a dysfunctional mitochondrial respiratory chain other sources plays a role in the development of atherosclerosis and its complications [40–42], which have been attributed to ROS-mediated vascular signaling pathways [17, 18]. In the present study, fluvastatin activated Nrf2 in VSMCs and this markedly increased ARE-driven promoter activity (Fig 2C) and the upregulation of ARE-targeted antioxidant genes (NQO1 and HO-1). These findings provide first evidence fluvastatin exerts its antioxidant effects via the ERK5-dependent activation of the Nrf2 pathway in vascular cells [17–19, 43].

It has been recently demonstrated AGEs and their receptor-ligand interactions play key roles in neointimal formation after vascular injury [28]. In fact, AGE-induced VSMC proliferation and ROS production are emerging as important mechanisms of atherosclerosis. Before commencing the present study, we hypothesized fluvastatin might reduce AGE-induced proliferation, and thus, we sought to identify the mechanism underlying the involvement of fluvastatin in AGE-induced cellular signaling. Treatment with AGEs increased the protein levels of the proliferation markers, cyclin D1 and CDK4, and markedly decreased p27 level, and these effects of AGEs were lost when ERK5 expression was depleted or inhibited by BIX02189 (Fig 5A). Our MTT assay and cell counting results show the antioxidant effects of fluvastatin are largely dependent on the ERK5-dependent Nrf2 pathway, because Nrf2 siRNA abolished their antioxidant cytoprotective responses. In addition, the overexpression of CA-MEK5α using an adenoviral system inhibited AGE-induced cell proliferation and cell cycle progression, and these effects were prevented by Nrf2 depletion (Fig 4 and Fig 6), which indicates ERK5 activation regulates cell cycle arrest via a Nrf2-dependent pathway.

NQO1 and HO-1 have been suggested as a downstream target of Nrf2 in inhibition of vascular smooth muscle cell proliferation through cell cycle arrest [44–46]. In this study, our data showed that statin induced Nrf2 and its target genes including NQO1 and HO-1 in an ERK5-dependent manner. In addition, inhibition of ERK5 and Nrf2 failed to inhibit fluvastatin-induced protein regulation of cell cycle-related genes including cyclin D1, CDK4, and p27 suggesting that ERK5-Nrf2 signal nodule regulates G1 cell cycle arrest in vascular smooth muscle cells.

A growing body of evidence suggested that ERK5 has cytoprotective effects via multiple pathways. Based on the genetic approach with gene knock out, it has been established that ERK5 is essential for endothelial viability and protects vascular leakage [47]. In addition, laminar shear stress protects endothelial cells from oxidative stress and inflammatory responses via ERK5 activation. However, laminar shear stress reduces endothelial cell proliferation an ERK5-dependent manner. On the other hand, ERK5 can be activated by many growth factors that are important in vascular smooth muscle cell proliferation. It is not clear whether growth factor-mediated ERK5 activation could regulate Nrf2 or not. ERK5 is not only a kinase but also a transcriptional coactivator with C-terminal transactivation domain, which is a unique characteristics compared to ERK1/2. It has been reported that statin induces both phosphorylation and transcriptional activation of ERK5 [48]. Therefore it is reasonable to pursue the molecular mechanism of ERK5-Nrf2 signal nodule in aspect of transcriptional regulation regarding the ambiguity of ERK5.

This study provides a molecular basis for the protective effects for statins. In particular, our finding regarding their induction of the ERK5-dependent regulation of cell proliferation and cell cycle arrest in VSMCs could eventually contribute to the development of a treatment for cardiovascular diseases. This study shows fluvastatin exerts induces antioxidant enzymes via an ERK5-dependent Nrf2 pathway.
